# Emerging role of STING signalling in CNS injury: inflammation, autophagy, necroptosis, ferroptosis and pyroptosis

**DOI:** 10.1186/s12974-022-02602-y

**Published:** 2022-10-04

**Authors:** Xinli Hu, Haojie Zhang, Qianxin Zhang, Xue Yao, Wenfei Ni, Kailiang Zhou

**Affiliations:** 1 Department of Orthopaedics, The Second Affiliated Hospital and Yuying Children’s Hospital of Wenzhou Medical University, Wenzhou, 325027 China; 2 Zhejiang Provincial Key Laboratory of Orthopaedics, Wenzhou, 325027 China; 3 Department of Orthopedics, Xuanwu Hospital of Capital Medical University, 45 Changchun Street, Xicheng, Beijing, 100053 People’s Republic of China; 4 The First Affiliated Hospital of Wenzhou Medical University, Wenzhou, 325000 Zhejiang China; 5 Department of Cardiology, Zhejiang Yuhuan People’s Hospital, Yuhuan, 317600 Zhejiang China; 6 Tianjin Key Laboratory of Spine and Spinal Cord, Department of Orthopaedics, Tianjin Medical University General Hospital, Tianjin, 300050 China

**Keywords:** STING, cGAS, Inflammation, Central nervous system injury, Cell death

## Abstract

Stimulator of interferons genes (STING), which is crucial for the secretion of type I interferons and proinflammatory cytokines in response to cytosolic nucleic acids, plays a key role in the innate immune system. Studies have revealed the participation of the STING pathway in unregulated inflammatory processes, traumatic brain injury (TBI), spinal cord injury (SCI), subarachnoid haemorrhage (SAH) and hypoxic–ischaemic encephalopathy (HIE). STING signalling is markedly increased in CNS injury, and STING agonists might facilitate the pathogenesis of CNS injury. However, the effects of STING-regulated signalling activation in CNS injury are not well understood. Aberrant activation of STING increases inflammatory events, type I interferon responses, and cell death. cGAS is the primary pathway that induces STING activation. Herein, we provide a comprehensive review of the latest findings related to STING signalling and the cGAS–STING pathway and highlight the control mechanisms and their functions in CNS injury. Furthermore, we summarize and explore the most recent advances toward obtaining an understanding of the involvement of STING signalling in programmed cell death (autophagy, necroptosis, ferroptosis and pyroptosis) during CNS injury. We also review potential therapeutic agents that are capable of regulating the cGAS–STING signalling pathway, which facilitates our understanding of cGAS–STING signalling functions in CNS injury and the potential value of this signalling pathway as a treatment target.

## Introduction

The central nervous system (CNS), which is composed of the brain and the spinal cord, is highly sensitive to external mechanical damage. Acute CNS injury, which mainly includes traumatic brain injury (TBI), spinal cord injury (SCI), subarachnoid haemorrhage (SAH) and hypoxic–ischaemic encephalopathy (HIE), is a leading cause of death and disability worldwide [1–4]. Acute CNS damage is associated with a tremendous social and economic expenditure and costs the medical system across the world more than US $200 billion each year [5]. Clinically, the conventional neuroprotective therapies for CNS injury mainly attempt to relieve mechanical compression by surgery combined with hyperbaric oxygen therapy, high-dose methylprednisolone, nerve dehydration and other comprehensive programmes [6–8]. Basic research has revealed potential treatments such as growth factors, tissue engineering, cell transplantation and neuroinflammation inhibitors [9–11], but major breakthroughs have not yet been achieved. Although these therapeutic measures alleviate the loss of neurological function to a certain extent, the long-term prognosis of CNS injury and the recovery of neurological function are still not optimistic. CNS injury is characterized by two temporal and spatial developments, including primary injury and secondary injury. Primary injury occurs when damage occurs and includes the cutting/tearing/extension of axons [12]. The primary physical lesion causes cell strain and membrane injury, which results in an imbalance of ions, the release of excitant amino acids, and oxidative species generation in the injured region [13, 14]. These processes trigger secondary injury that jointly extend the damage to healthy adjacent cells, leading to inflammation and neuronal cell death and eventually to loss of function [15]. Hence, apoptosis and subsequent inflammatory processes are prime biological mechanisms underlying CNS damage. Identifying methods for regulating neuroinflammation to alleviate the death of nerve cells is key in the treatment of CNS injury. The disruption of cellular homeostasis could induce cumulative cytoplasmic DNA, such as DNA lesions, disrupted mitochondria and exosomes, in which cyclic guanosine monophosphate-adenosine monophosphate synthase (cGAS) senses and is stimulated with the combination of double-stranded DNA (dsDNA) [16, 17]. Specifically, cGAMP and other cyclic dinucleotides (CDNs) propagate the signal to the endoplasmic reticulum (ER) protein called stimulator of interferons genes (STING). STING was first described as a protein that interacts with major histocompatibility complex class II molecules, but the relevance of this interaction remains unclear [18]. To further determine the origin of proteins that overexpress interferon-β (IFN-β), Ishikawa et al. employed an expression screening system to identifying proteins able to induce interferon-β (IFN-β) secretion, and in the study, approximately 5500 human and 9000 murine full-length cDNAs were individually transfected into cells harbouring a luciferase gene under the control of the IFNβ promoter [19]. Five genes whose overexpression led to significant induction of IFNβ were found, and one of the previously uncharacterized molecules is denoted STING by the authors [19]. Subsequent study of STING-deficient mice confirmed the essential role of STING in innate responses to stimulate IFNβ [20]. STING dimerizes and translocates from the ER to perinuclear structures, such as the Golgi apparatus. STING binds to TANK-binding kinase 1 (TBK1), which results in its phosphorylation. Phosphorylated STING then binds to positively charged surfaces of interferon regulatory factor 3 (IRF3), which leads to its phosphorylation and activation by TBK1 [21]. The phosphorylation of IRF3 induces the translocation of IRF-3 from the cytoplasm to the nucleus. IRF-3 binds to the IFN-stimulated response element of the IFN-stimulated gene 15 (ISG15) promoter and increases its transcriptional activation [22]. Afterward, the signal peaks in interferon regulatory factor 3 (IRF3) and NF-κB targets, causing IFN secretion [23]. Furthermore, some evidence verifies the significance of IFN in neuroinflammation and cell death, which implies the disruption of IFN responses in different immunity-regulated disorders, such as CNS injury [24]. It has been revealed the essential role of cGAS–STING, which signals a primary inducing factor of IFNs for cytosolic DNA or CDNs [25]. Nevertheless, whether the cGAS/STING/IFN axis facilitates the pathogenesis of CNS injury still needs investigation. Our research initially discusses the cGAS–STING pathway and studies targeting the participation of STING in CNS injury. Moreover, we examine the functions of the cGAS–STING pathway in the IFN immune response and certain cell death pathways, such as autophagy, necroptosis, ferroptosis and pyroptosis. Additionally, we highlight the molecular mechanisms and biological roles of cGAS–STING pathway activation to reinforce the biotherapeutic validity of cGAS–STING in CNS damage. We ultimately aim to provide a more in-depth understanding of the mechanism through which STING signalling modulates the nerve inflammatory response in CNS injury and thus reveal the underlying therapeutic value of the cGAS–STING pathway in acute CNS damage.

## STING signalling and cGAS–STING pathway

**Fig. 1:**
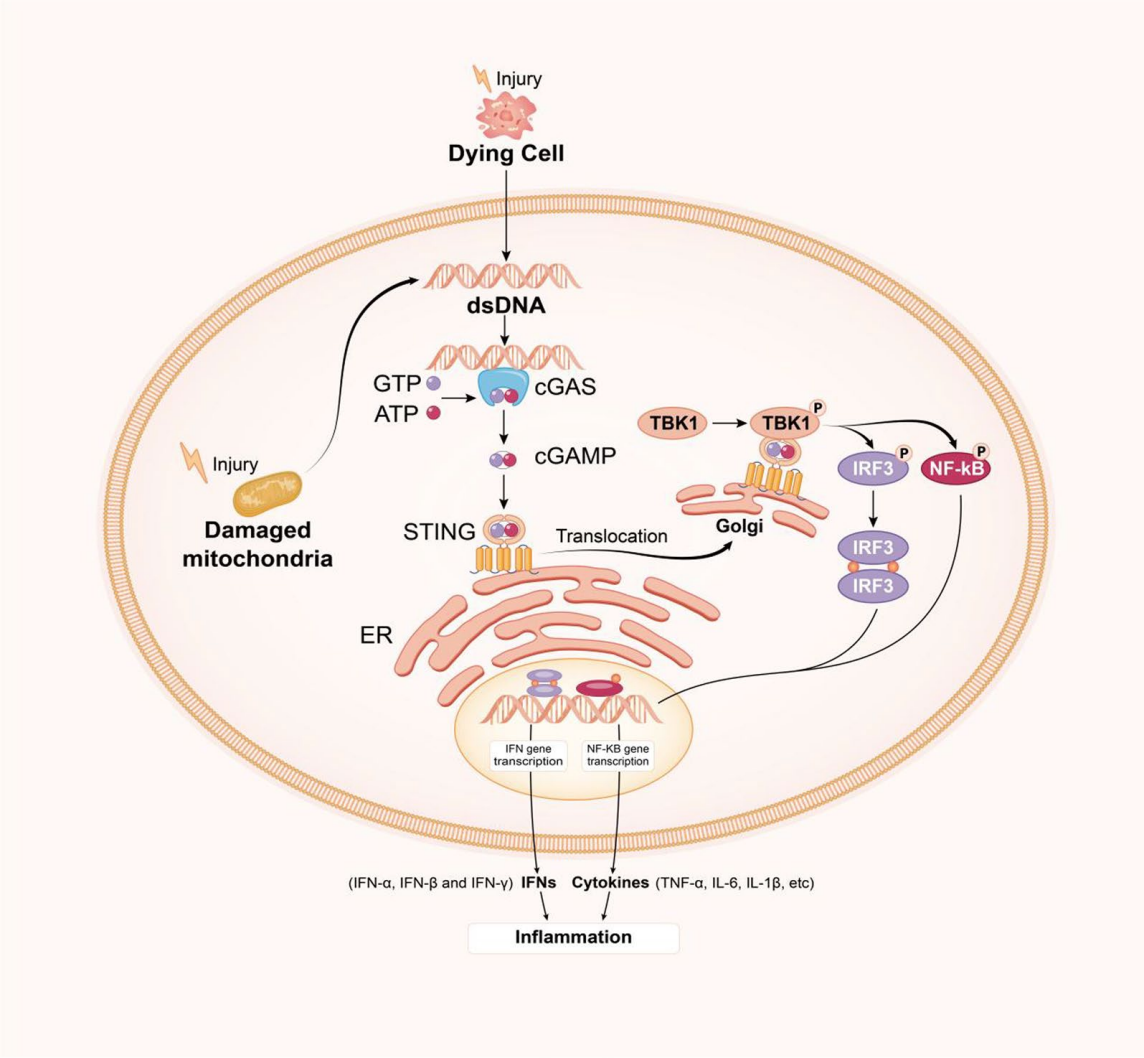
cGAS–STING signalling pathway. Cytosolic DNA (either foreign or self) is recognized by cGAS, which converts ATP and GTP into the second messenger 2′,3′-cGAMP, and this messenger binds and activates STING located in the endoplasmic reticulum. STING translocates to the Golgi, where it recruits and induces the phosphorylation of IRF3 and IKK via TBK1. Cytosolic IRF3 dimerizes and enters the nucleus after phosphorylation, which results in induction of the transcription of IRF3 target genes and the release of type 1 IFNs. Parallel to IRF3 activation, STING also activates IKK, which induces the transcription of NF-κB transcription factors and the release of cytokines

As stimuli, DNA disruption, mitochondrial damage, apoptosis, exosomes, DNA viruses, retroviruses and microbes facilitate the generation of pathogen-related molecular patterns (PAMPs) and danger-related molecular patterns (DAMPs). PAMPs or DAMPs can be identified via pattern-recognition receptors (PRRs), which serve as innate cellular sensors that induce a cellular stress response. In eukaryotes, hereditary DNA substances are limited to the nucleus and mitochondria, whereas cytosolic or extracellular DNA serving as PAMPs activates DNA sensors to induce innate immune responses [26]. To offset these deleterious signals, cells have different DNA sensors, such as Toll-like receptor 9 (TLR9), absent in melanoma 2 (AIM2), cyclic GMP-AMP synthase (cGAS), interferon gamma-inducible protein 16 (IFI16), DNA-dependent activators of IRFs, IFI16, and RNA polymerase III [27, 28], and among these, only TLR9, AIM2, and cGAS have been satisfactorily elucidated, whereas the others remain unvalidated [29]. TLRs are expressed on the plasma and endosomal membranes of immune cells and serve as sensors of exterior and intrinsic signals that endanger the host [30]. In contrast, DNA in the cytosolic compartment is identified via two primary receptors: AIM2 and cGAS. AIM2 falls within the category of the PRR family and forms inflammasomes, where activated caspase-1 in the presence of enormous signalling multiprotein oligomers induces the maturation of pro-interleukin-1β (IL-1β) and pro-interleukin-18 (pro-IL-18) by proteolytic cleavage and triggers inflammation via pyroptosis [31]. In comparison, for cytosolic DNA, cGAS primarily activates the generation of IFNs. In contrast to the majority of other PRRs, cGAS does not directly generate a signalling platform at the molecular level but rather activates the generation of an inherent second messenger, which is identified by receptors [32]. Cytosolic dsDNA is recognized by cGAS and activates immunity. The combination of cGAS with cytosolic DNA stimulates cGAS via conformational variations and dimerization, which induces catalytic site rearrangement [33, 34]. Binding does not depend on the DNA sequence but relies instead on the DNA length [35]. The binding of DNA to cGAS causes conformational variations in cGAS, which activates the generation of 2′,3′-cyclic AMP-GMP (2′,3′-cGAMP), a CDN with a unique phosphodiester linkage that uses adenosine triphosphate (ATP) and guanosine triphosphate (GTP) [36]. In its resting state, STING associates with the ER-resident protein sensor stromal interaction molecule (STIM1), which facilitates its localization in the ER [37]. STING binding to cGAMP dissociates the binding between STIM1 and STING and redirects the binding of STING to SEC24C, a constituent of coat protein II (COPII). This step induces the translocation of STING from the ER to the Golgi apparatus or endosomes and autophagy-associated compartments via the ER–Golgi intermediate compartment (ERGIC) [38, 39]. During this activity, STING recruits TBK1 to its C-end tail and facilitates TBK1 autophosphorylation, which stimulates the phosphorylation of IRF3 [40, 41]. Moreover, NF-kB activates STING cascades. Consequently, these factors enter the nucleus and work jointly to trigger the generation of type I IFNs [19]. Type-I IFNs signal through the Janus kinase (JAK)-signal transducer and activator of transcription (STAT) pathway to elicit an immunostimulatory response through interferon-stimulated gene (ISG) induction [42, 43], which results in the secretion of proinflammatory cytokines and chemokines, including tumour necrosis factor-α (TNF-α), interleukin-6 (IL-6), interleukin-1β (IL-1β) and the type-I IFNs themselves (IFN-α, IFN-β and IFN-γ) [44] (Fig. 1).

## STING signalling in CNS injuries

### STING activity in TBI

Increasing evidence shows that neuroinflammation facilitates nerve damage in response to TBI [45, 46]. A prolonged or chronic neuroinflammatory response contributes to cell death, as observed in animal studies [47]. IFNs serve as key inflammatory regulators that are needed to mount an inflammatory response to TBI in the brain [48]. A reduction in type-1 IFN signalling decreases neuroinflammation and reduces damage in a controlled cortical impact (CCI) mouse model [24]. Furthermore, Abdullah et al. first discovered a key role for STING as a mediator in IFN generation and the nerve inflammatory response after TBI. These researchers observed that STING^−/−^ mice exposed to CCI experience less damage than their wild-type littermates [49]. Such nerve protection could be partially due to decreased proinflammatory cytokines. Moreover, researchers have observed elevated STING mRNA levels in the human TBI cerebrum at autopsy, indicating a role for STING activation in TBI. Moreover, a previous study indicated interactions between the cGAS–STING pathway and autophagy machinery in TBI [49]. Inhibition of autophagic flux occurs after acute CNS injury. Phospholipases, sphingosine and ROS are produced and translocated to the lysosomal membrane after CNS injury and cause increases in lysosomal membrane permeability and lysosomal damage [50, 51]. Because lysosomal function can affect the fusion of autophagosomes with lysosomes, the decrease in lysosomal function after CNS injury leads to impairment of the autophagic clearance flux [52, 53]. Since the function of autophagy is dependent on its flux, autophagy is expected to contribute to STING activation in cases where flux is inhibited, such as moderate to severe TBI or most cases of SCI. In addition, Sen et al. reported an upstream STING catalyst in TBI, namely, protein kinase R-like ER kinase (PERK). These researchers showed excessive secretion of neuronal IFN in response to the activation of STING–TBK1–IRF3 signalling after abnormal PERK activation [54]. Remarkably, TBI-triggered STING activation is weakened in mice administered a PERK inhibitor (GSK2656157), which contributes to decreased brain injury and satisfactory injury recovery, and these findings emphasize the therapeutic value of GSK2656157 in TBI therapy [54]. In addition, another recent study found that GSK2656157 targets receptor-interacting protein kinase 1 (RIPK1), which mediates necroptosis. This finding suggests a potential relationship between STING and necroptosis [55]. In summary, STING signals play an important role in mediating brain injury, which is closely related to autophagy and programmed necrosis (as will be discussed in more detail later).

### STING activity in HIE

HIE is characterized by the accumulation of dead cells, disrupted inherent DNA metabolism, and DNA leakage into the cytosol [56]. Gamdzyk et al. found that STING notably increases with the expression of cGAS two and three days after HIE in the damaged area; these researchers also observed STING expression in microglia and astrocytes in the newborn rat cerebrum [23]. One study has also shown that the cGAS–STING pathway can be catalysed via long-interspersed element-1 (LINE-1) [57]. Stavudine is a famous anti-HIV-1 medicine that can suppress the reverse transcription of LINE-1 [58]. Gamdzyk et al. discovered that stavudine efficiently suppresses LINE-1 activity and reduces STING activation after HIE; hence, LINE-1 facilitates STING activation in response to HIE [23]. In addition, the activation of cGAS is potent in response to dsDNA induced by cell death, which promotes proinflammatory processes. A151, a cGAS antagonist, is a synthetic oligodeoxynucleotide that abrogates the activation of cytosolic nucleic acid-sensing cGAS and the AIM2 inflammasome by binding to these molecules in a manner that is competitive with immune-stimulatory DNA, which results in attenuation of brain injury in response to ischaemic stroke [17]. Even though the exact molecular process of cGAS in nerve inflammation after stroke remains unclear and requires more investigation, the cGAS–STING pathway surely participates in the response to CNS injury.

### STING activity in SAH

SAH, which is primarily induced by ruptured intracranial aneurysms, remains a remarkable clinical challenge with prominent incidence and death rates worldwide [59]. Recently, Peng et al. found that the STING levels are notably increased 12 h hours postinjury, peaked within one day, and then progressively decreased in a mouse model of SAH. Furthermore, through immunofluorescent staining, these researchers found that STING is primarily expressed in microglia rather than in neurons or astrocytes [60]. Because TBK1 is the immediate substrate downstream of STING and the activation and phosphorylation of TBK1 can be induced by STING, the phosphorylated TBK1 levels were examined to identify the efficacy of C-176 (STING antagonist) and CMA (a STING agonist) in blocking or catalysing STING [61]. The administration of CMA to SAH mice increases the p-TBK1/TBK1 ratio and exacerbates nerve injury and nerve activities compared with the results found for mice treated with vehicle. In contrast, the use of the STING suppressor C-176 just after SAH modelling induces nerve protection by notably reducing TBK1 phosphorylation [60]. Based on this evidence, microglial STING induces inflammation in response to SAH, whereas STING suppression partially weakens SAH-induced inflammatory damage. This study confirmed that STING is involved in SAH and is an underlying treatment target in this disease.

### STING activity in SCI

SCI is a destructive CNS injury involving primary and secondary injury [62, 63]. Primary injury is not reversible, whereas secondary damage is comparatively treatable [64, 65]. The main events in secondary damage include ischaemic symptoms, inflammation and cell death, among which inflammation is the crucial target [66]. At present, evidence shows that STING signalling pathways are pivotal for nerve disorders and SCI. Wang et al. demonstrated that protein and mRNA expression of STING is markedly induced in the progression of SCI [67]. On the one hand, STING ablation suppresses the proinflammatory IKKb/IkBa/NF-kB pathway, which notably decreases proinflammatory biomarker expression in SCI. On the other hand, mitogen-activated protein kinases (MAPKs), which regulate inflammation, including p38, ERK1/2 and JNK, are markedly weakened in mice overexpressing STING. These two factors ultimately show that STING facilitates the inflammatory response [67]. Moreover, STING exhibits a favourable impact on the activation of TBK1, which modulates IRF3 activation and NF-κB activation [68]. In summary, STING markedly exacerbates inflammatory events and nerve injury by directly binding to TBK1, which stimulates the NF-kB and MAPK signalling pathways. Hence, therapies interfering with the mutual effect of STING-TBK1 may represent a potential treatment for SCI.

### STING activity-associated cell type in CNS injury

Various types of CNS injury contribute to cell death and subsequent inflammation, and oxidative stress is an important contributor to the pathophysiology of a variety of CNS injuries, including SCI, TBI, SAH and HIE [69, 70]. Oxidative stress and the resultant accumulation of ROS can lead to a number of different DNA lesions [71]. DNA accumulation provokes neuroinflammation through aberrant activation of the cGAS–STING pathway [72, 73].

No study has investigated which nerve cells play a main role in the activation of cGAS–STING pathways. Many studies of CNS injury suggest that microglia are the principal innate immune cells in the brain and the first responders to pathological insults and produce high levels of type I IFN [74, 75]. Currently, most research focuses on the role of microglia in the activation of cGAS–STING pathways in CNS injury [76, 77]. Moreover, one study showed that astrocytic STING expression is widespread following TBI, whereas neuronal expression of STING is restricted near the site of injury [49]. The results suggest that neurons may play a weaker role than astrocytes and microglia in the STING-mediated response after CNS injury. To further compare the extent of the activation of the cGAS–STING pathway in neurons, astrocytes and microglia, a study on type I IFN and herpes simplex encephalitis was performed. Scholars found that the relative stimulation of type I IFN production by synthetic DNA in the three cell types can be ranked neurons < astrocytes < microglia [78]. Therefore, we assume that astrocytes and microglia may be the major cells involved in the STING-mediated response after injury.

## Interactions between STING signalling and different types of cell death

In addition to cytokine induction, STING signalling plays a role in cell death pathways, such as autophagy, necroptosis, ferroptosis and pyroptosis. Because STING is essentially involved in innate immunity, the induction of cell death might represent an ultimatum to prevent injury. However, excessive cell death results in loss of bodily function. Therefore, exploring the relationship between STING and cell death will provide more insight for the treatment of CNS injuries.

### Role of the STING pathway in autophagy in CNS injury

**Fig. 2:**
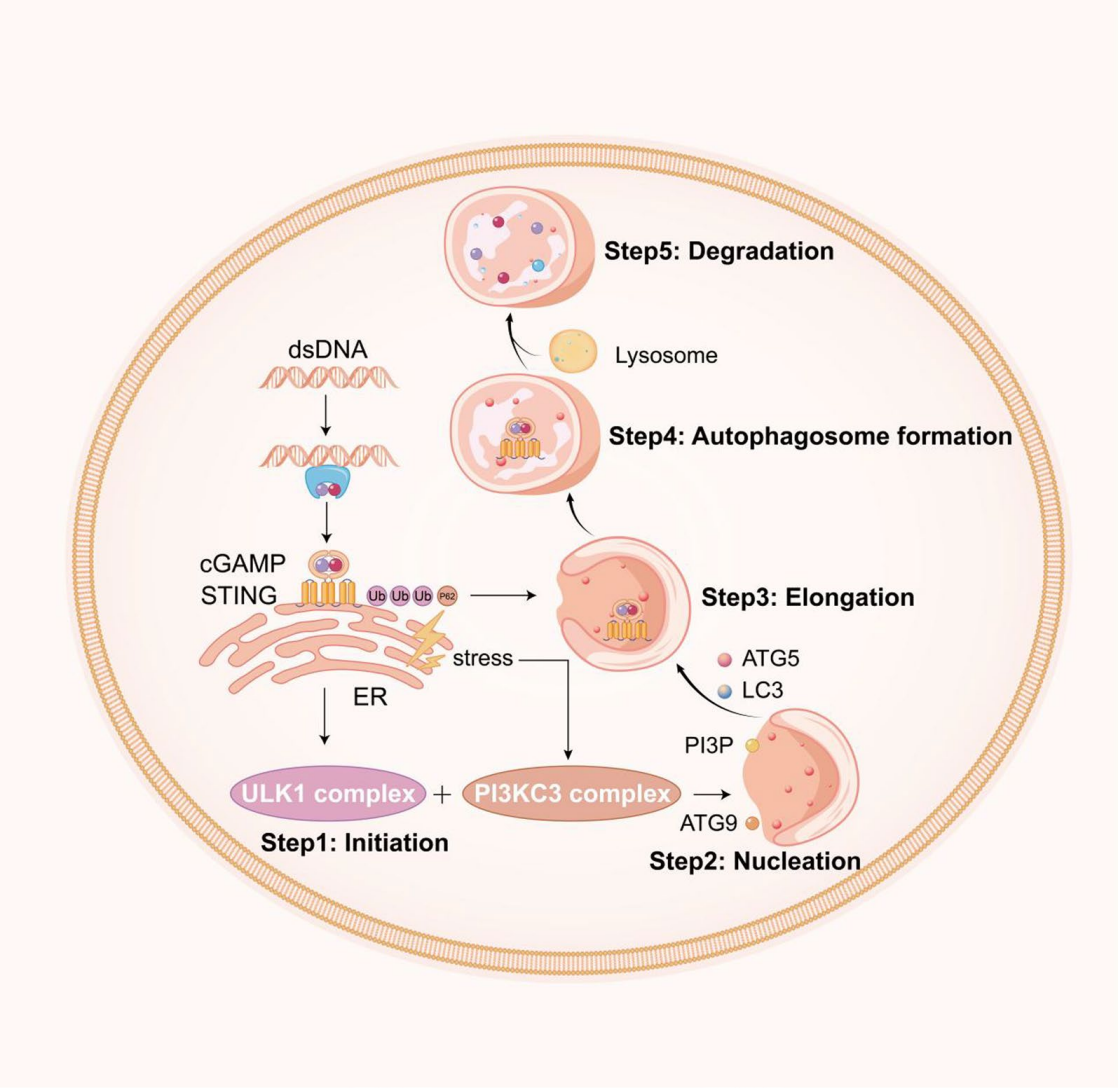
Complexity of interactions between the cGAS–STING signalling pathway and autophagy. The autophagy process involves five key steps: (1) initiation, (2) nucleation, (3) elongation, (4) autophagosome formation, and (5) degradation. cGAS–STING activation can initiate autophagy and the subsequent five key steps, which triggers its own degradation. After autophagy initiation, cGAS–STING is ubiquitinated and binds p62. These factors are then packaged into autophagosomes and terminally sorted to lysosomes, and each step is regulated by specific ATG proteins, as highlighted, or explicit proteins, such as the ULK1, Beclin-1, P13P, and LC3 conjugation systems

Autophagy, a fundamental cellular process in eukaryotes that involves the sequestration of cytosolic constituents within double membrane-bound autophagosomes that are subsequently fused with endolysosomal vesicles, which leads to the degradation and recycling of the sequestered substrates, plays an important role in both the activation and regulation of innate and adaptive immune responses [79]. The literature details evidence showing increases in autophagy markers after TBI, and both protective and detrimental effects have been observed. This double-edged sword of autophagy reported after brain trauma may be due to lack of an understanding of its mechanisms and cell-type specificity within the CNS. Recently, a role for the STING and IFN pathways in autophagy has also been proposed [80–82] (Fig. 2). p62 is part of a larger family of ubiquitin-binding autophagy receptors that link ubiquitin and autophagy by harbouring both a ubiquitin-binding domain and an LC3 (light chain 3)-interaction region. Prabakaran et al. reported that the ubiquitin-binding selective autophagy protein p62/SQSTM1 is essential for the DNA- and cGAMP-stimulated degradation of STING. STING is ubiquitinated through a K63 linkage and is recruited to p62-positive compartments [83]. STING is not degraded in p62-deficient cells, which produce elevated levels of IFN and IFN-stimulated genes (ISGs). Therefore, p62 is essential for targeting STING for autophagosomal degradation following stimulation of the cGAS–STING pathway [83]. STING undergoes ubiquitination and is packaged into autophagosomes with the help of p62 to be terminally sorted into lysosomes [84]. cGAS or STING is digested immediately in the autophagolysosome after transient activation of downstream signalling [85]. Autophagy functions as a negative feedback loop that ensures transient cGAS–STING signalling and avoids consistent overactivation of the pathway. Thus, the impairment of autophagy may give rise to destructive inflammatory diseases. Liu et al. found that the binding and dimerizing activities of cGAMP are indispensable for STING-induced autophagy. These researchers also showed that autophagy-related genes (ATGs), such as ATG5 and ATG16L1, plays an active role in the STING–TBK1 interaction [86]. Mutants that abolish STING dimerization and cGAMP binding diminish the STING–LC3 interaction and subsequent autophagy, which suggests that STING activation is indispensable for autophagy induction. Other research suggests that the observed increases in the LC3-II and p62 levels are not an indicator of impaired autophagic flux but rather an indicator of enhanced autophagic activity, which serves as a protective mechanism to reduce cellular damage following TBI. In summary, autophagy proteins downregulate STING activity through both canonical and noncanonical pathways, which may represent a means of avoiding excess inflammation. TBK1 has also been reported to function upstream of NF-κB activation [87]. In concert, these transcription factors induce antiviral and proinflammatory gene expression. Downstream of STING, TBK1 also triggers autophagy independent of its function of inducing gene expression [88]. In this context, STING colocalizes with markers of autophagosomes at late stages following activation [39]. In the future, additional studies should focus on understanding the structural basis of the STING–TBK1 interaction for immune activation and the STING–LC3 interaction for autophagy induction.

### Role of the STING pathway in necroptosis in CNS injury

**Fig. 3:**
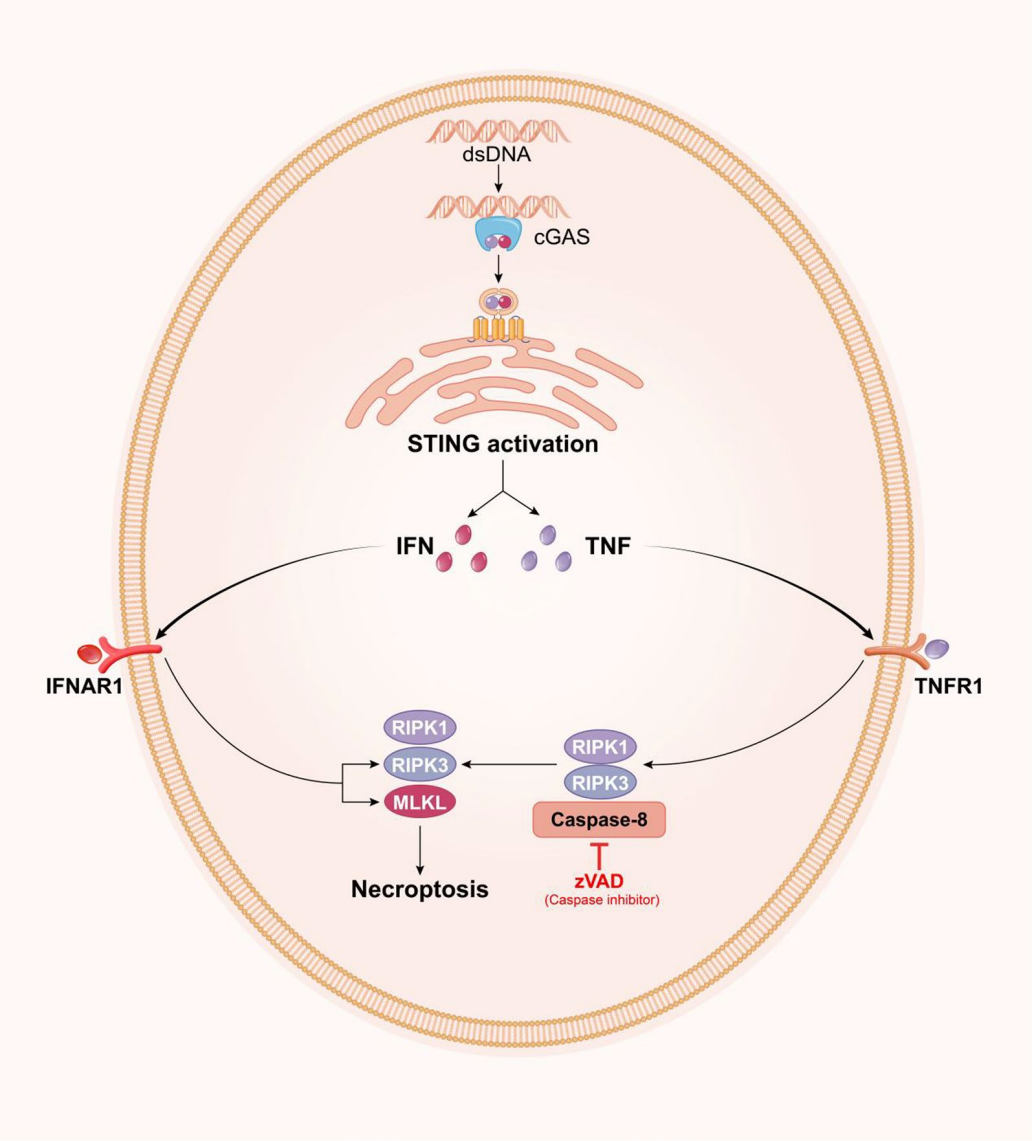
Activation of necroptosis by cGAS–STING signalling. Initiation of necroptosis by cGAS–STING signalling. Activation of the cGAS–STING pathway by mitochondrial DNA results in the production of IFN and TNF. The binding of IFN to IFNAR1 and TNF to TNFR1 results in RIPK1–RIPK3 activation when caspase-8 is inhibited. RIPK1–RIPK3 activate MLKL to execute necroptosis. Moreover, IFN upregulates RIPK3 and MLKL expression

Necroptosis is a form of necrotic cell death that occurs downstream of receptor-interacting protein kinase (RIPK)1 and 3 activation and disruption of the plasma membrane by the pseudokinase mixed lineage kinase like-domain (MLKL) [89, 90]. IFNs regulate host immune responses by binding the receptor and activating STAT1/2 transcription factors to regulate a diverse family of genes termed ISGs [91]. DNA, possibly from DNA damage repair or mitochondrial stress, activates the cGAS–STING pathway, which leads to constitutive IFN production that feeds back onto cells to sustain the expression of many ISGs. MLKL is an ISG that must be sufficiently expressed to facilitate oligomerization and cell death [92]. In addition, Brault et al. showed that following DNA detection, the cGAS–STING pathway triggers necroptosis in primary macrophages when caspases are suppressed [93]. Notably, this cell death response requires STING-dependent production of both IFN and TNF, and the induction of necroptosis by STING activation involves reciprocal and synergistic signalling by these two pathways. Furthermore, necroptosis triggered by IFN signalling in the absence of pathogen-associated ligands requires tumour necrosis factor (TNF) signalling despite a lack of TNF induction upon interferon treatment. Future studies will assess the role of these two pathways in the induction of necroptosis, in which both IFN and TNF signalling are critical for pathogen clearance (Fig. 3).

### Role of the STING pathway in ferroptosis in CNS injury

**Fig. 4:**
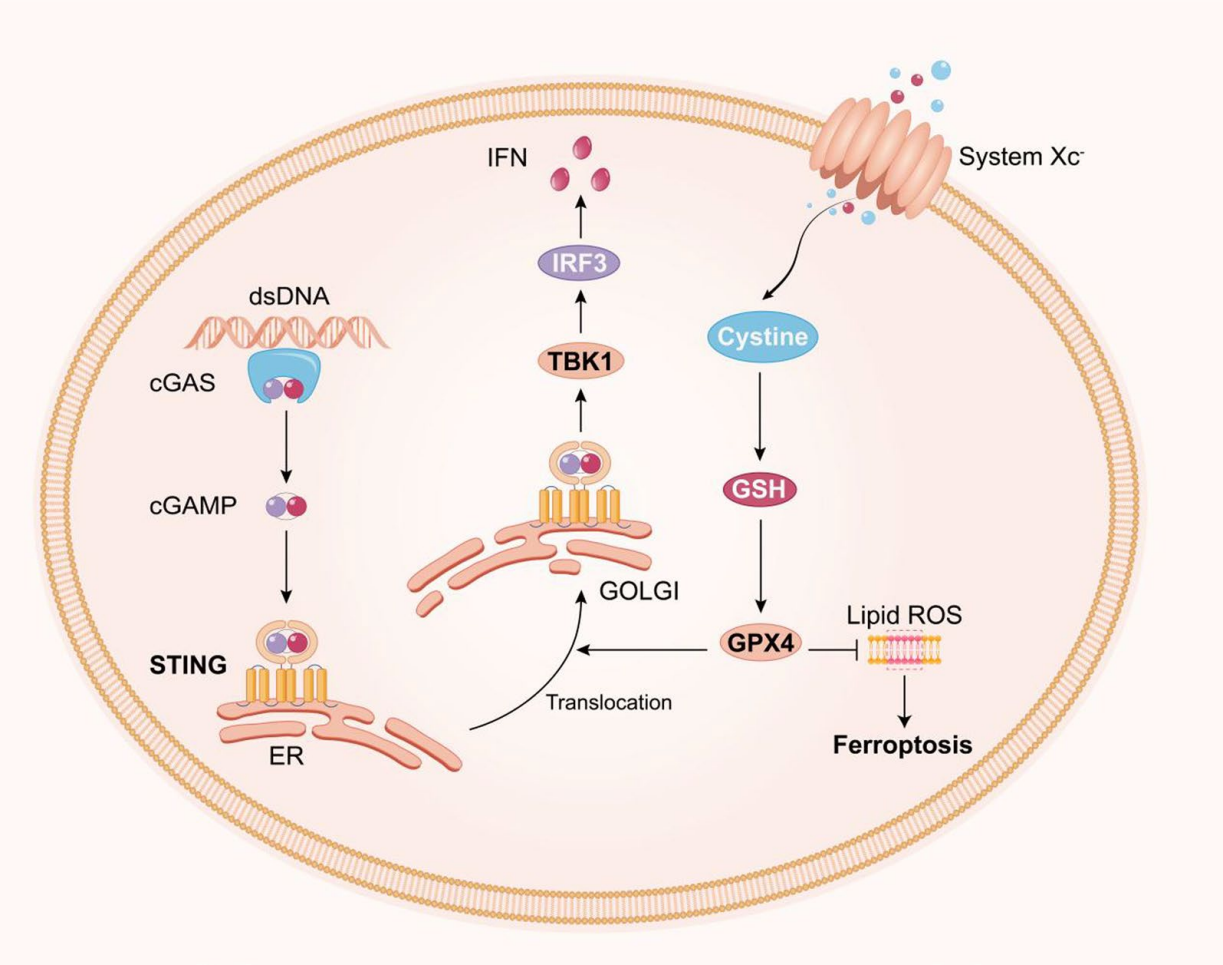
Interactions between the cGAS–STING pathway and ferroptosis. Loss of GPX4 function, either directly or indirectly, is currently thought to be the key event leading to the onset of ferroptosis. In addition, GPX4 is needed for activation of the cGAS–STING pathway. GPX4 deficiency enhances lipid peroxidation, which promotes STING carbonylation and inhibits its translocation from the ER to the Golgi complex

Ferroptosis is a newly discovered form of programmed cell death that results from the accumulation of iron-dependent lipid peroxide, and the term ‘ferroptosis’ was first used by Stockwell et al. in 2012 [94]. Ferroptosis is genetically, morphologically and biochemically distinct from apoptosis [95]. The amino acid transport system Xc (System Xc) is a cystine/glutamate antiporter. Intracellular cysteine is reduced to cysteine for the biosynthesis of GSH. Glutathione peroxidase 4 (GPX4), a member of the GPX family, is also involved in ferroptosis. GPX4, together with glutathione (GSH), reduces free hydrogen peroxide (H_2_O_2_) or organic peroxide (ROOH) into water or their corresponding alcohols. GSH depletion deactivates GPX4 and thus elevates the lipid ROS levels. Recent evidence demonstrates that GPX4 is needed for activation of the cGAS–STING pathway. GPX4 deficiency enhances lipid peroxidation, which promotes STING carbonylation and inhibits its translocation from the ER to the Golgi complex [96]. Overall, these findings suggest a connection between ferroptosis and the cGAS–STING pathway (Fig. 4). Although ferroptosis is an emerging pathogenic factor in CNS injury [97–99], the mechanism underling the involvement of the cGAS–STING pathway in ferroptosis in CNS injury remains unclear. Obtaining a better understanding of the contributions of the cGAS–STING pathway to ferroptosis is important for improving the therapeutic strategies used for patients with CNS injury in the near future.

### Role of the STING pathway in pyroptosis in CNS injury

**Fig. 5:**
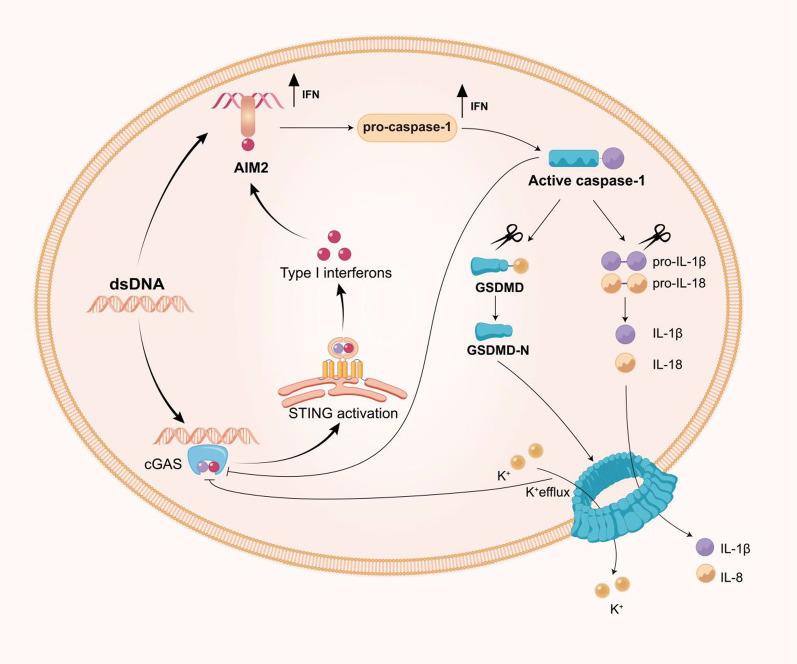
Interplay between the cGAS–STING signalling pathway and pyroptosis. The AIM2 inflammasome activates caspase-1, which activates IL-1β and triggers pyroptosis. On the one hand, cGAS-mediated IFN responses increase caspase-1 expression, which in turn increases the release of IL-1β and pyroptotic cell death. On the other hand, the AIM2 pathway counteracts cGAS–STING signalling. First, cGAS is a target for caspase-1 cleavage. Second, gasdermin D activated by caspase-1 leads to potassium ion (K^+^) efflux, which inhibits cGAS. In addition, the cGAS–STING pathway triggers the NLRP3 inflammasome through several mechanisms, and this process lags behind canonical interferon signalling

The term pyroptosis was first observed in macrophages that underwent unique caspase-1 programmed cell death following exposure to Salmonella [100]. In brief, inflammasomes are large multiprotein complexes composed of germline-encoded pattern-recognition receptors of the Nod-like receptor (NLR) family (NLRP1; NLRP2; NLRP3; NLRC4 and AIM2), adaptor protein apoptosis-associated speck-like protein (ASC) and pro-caspase-1 [101, 102]. In response to pathogenic or physiological perturbations in the cytosol, pro-caspase-1 and ASC recruit NLRP3 or AIM2 to form inflammasomes. Subsequently, pro-caspase-1 is cleaved to form caspase-1, which not only promotes cleavage of pro-IL-1β/18 but also cleaves gasdermin D (GSDMD) into two fragments [103]. The N-terminal fragment forms 10–15-nm pores in the cell membrane, which eventually leads to the discharge of inflammatory factors, cell swelling, and membrane rupture [104]. A considerable body of literature highlights the critical role of the AIM2 inflammasome in host defence mechanisms [105]. One study demonstrated that cGAS–STING–IFN1 pathway activation increases the AIM2 protein levels to induce a robust innate immune response [106]. Similarly, cGAS-mediated IFN responses upregulate caspase-1 and caspase-11 expression, which in turn increases IL-1β release and pyroptotic cell death [107]. These observations indicate that AIM2 activation and STING signalling amplify these responses. However, Banerjee et al. discovered that GSDMD pore formation results in potassium ion efflux to inhibit the dsDNA-mediated activation of the cGAS–STING pathway and thus result in reduced IFN production. In the absence of GSDMD-mediated potassium ion efflux, the binding of dsDNA to cGAS is enhanced and exacerbates the damage-inducing IFN response [108]. Additionally, caspase-1 limits the cytosolic DNA-mediated activation of cGAS–STING by directly cleaving cGAS during canonical inflammasome activation [109] (Fig. 5). One possible explanation is that the innate immune system maintains a balance between the expression and/or activation of different DNA sensors to prevent overactivation of inflammatory responses. Collectively, these studies highlight the sophisticated interplay between cGAS–STING and inflammasome components that could be leveraged to prevent hyperinflammation.

### Potential interrelationship among cell death pathways acting downstream of STING signalling

Lysosomes act as cell recycling centres and are filled with numerous hydrolytic enzymes that can degrade most cell macromolecules. The permeabilization of the lysosomal membrane and the subsequent leakage of the lysosomal content into the cytoplasm results in cell death. One study has revealed that STING signalling leads to the deceleration of lysosomal digestion by affecting the regulation of the lysosomal pH. Continuous STING pathway activation contributes to termination of the autophagic flux by perturbing lysosomal digestion [110]. The autophagy–lysosomal pathway plays an essential role in cellular homeostasis as well as a protective function against a variety of diseases, including neurodegeneration. Conversely, the inhibition of autophagy due to, for example, lysosomal dysfunction can lead to the pathological accumulation of dysfunctional autophagosomes and consequent neuronal cell death [111]. Cytoplasmic phospholipase A2 is activated after CNS injury, which can damage lysosome cellular membranes and then lead to leakage of lysosomal cysteine protease cathepsin B (CTSB) [112]. In addition, CTSB may aggravate cell death by activating the NLRP3 inflammasome and promoting caspase-1-induced pyroptosis [113]. Another study showed that leakage of CTSB is an important molecular event that mediates organelle-specific initiation of ferroptosis. Mechanistically, nuclear CTSB accumulation causes DNA damage and subsequent activation of the stimulator of interferon response cGAMP interactor 1 (STING1/STING)-dependent DNA sensor pathway, which ultimately leads to ferroptosis [114]. Lysosomal membrane permeabilization (LMP) plays an indispensable role in the regulation of necroptosis [115].

No study has defined the interrelationship among cell death pathways acting downstream of STING signalling. Based on the aforementioned research, we assume that lysosomes may be an important link among autophagy, pyroptosis, ferroptosis and necroptosis acting downstream of STING signalling. Further studies are needed to determine whether this mechanism occurs in CNS injury.

## cGAS–STING pathway as a therapeutic target in CNS injury

Sterile inflammation induced by the cGAS–STING pathway plays an important role in CNS injury. Thus, inhibitors of the cGAS–STING pathway may represent potential targets in the treatment of CNS injury. Previous studies have indicated that damage to cells may abnormally discharge DNA from the cytoplasm and that cGAS catalyses the production of 2′,3′-cGAMP by recognizing DNA from the cytoplasm. Subsequently, 2′,3′-cGAMP transmits signals to downstream STING, which induces transcription factors such as IRF3 and NF-κB to translocate into the nucleus and express inflammatory factors such as IFN. This process can activate both innate immunity and adaptive immunity [116]. Therefore, endogenous or exogenous DNA combining with and activating cGAS and STING activation are both significant factors, and these findings provide hope for the treatment of CNS injury by inhibiting these two links. In the past, many medicines have been proven to have inhibitory functions in the cGAS–STING pathway and have provided viable therapeutic methods for some diseases, such as autoimmune disease, pancreatitis and infectious disease (Table 1). However, in the field of CNS injury, the function of inhibitors in the cGAS–STING pathway remains unknown.

**Table 1:** Inhibitors of the cGAS–STING pathway

Target	Inhibitor(s)	Mechanism	Disease	References
Cytosolic DNA	Metformin or rapamycin	Autophagy can decrease the load of cytoplasmic DNA	Ageing-associated inflammation	[134]
cGAS	RU.521	Inhibits the activity of cGAS by binding the catalytic sites in cGAS to influence the synthesis of 2’,3’-cGAMP	Not mentioned	[117]
	A151	Abrogates the activation of cytosolic nucleic acid-sensing cGAS and the AIM inflammasome by binding to these molecules in a manner that competes with immune-stimulatory DNA	Middle cerebral artery occlusion	[17]
	Quinacrine	Prevents the combination of cGAS and DNA and thus inhibits the cGAS–STING signalling pathway		[118]
	Aspirin	Acetylates cGAS at three lysine residues and blocks cGAS activity	Aicardi–Goutieres syndrome	[119]
	Epigallocatechin gallate	Blocks the interaction between cGAS and DNA by inhibiting the activity of GTPase-activating protein SH3 domain-binding protein 1 (G3BP1)	Not mentioned	[120]
STING	C-176 and H151	Combines with CDN by integrating with Cys-91 sites in rat STING, which blocks the palmitoylation of STING		[61]
	Carbonyl cyanide 3-chlorophenylhydrazone (CCCP)	Disrupts the mitochondrial membrane potential, which leads to repressed communication between STING and TBK-1	Not mentioned	[129]
	Compound 18	Binds to STING and reduces the binding affinity of cGAMP to STING	Not mentioned	[131]
	AstinC	Targets the downstream transcription factor IRF-1	Not mentioned	[132]
IFNs	Ruxolitinib	Electively inhibits the IFN-γ/JAK/STAT signalling pathway	Traumatic spinal cord injury	[137]
	Baricitinib	Blocks IFN signalling	Chronic atypical neutrophilic dermatosis with lipodystrophy and elevated temperatures Stimulator of IFN genes-associated (STING-associated) vasculopathy with onset in infancy	[138]

### Inhibitors of cGAS

cGAS catalyses GTP and ATP to generate 2′,3′-cGAMP after recognizing endogenous and exogenous DNA, which represents a major facet of the cGAS–STING pathway. Therefore, preventing the combination of cGAS and DNA and inhibiting the catalytic activity of cGAS constitute a feasible method for blocking this pathway. By establishing methods for monitoring cGAS activity in vitro, Vincent et al. screened a compound called RU.521, which specifically inhibits the activity of cGAS by binding the catalytic sites in cGAS to influence the synthesis of 2′,3′-cGAMP [117]. In addition, Qian et al. found that an oligodeoxynucleotide called A151, which contains the immunosuppressive motif *TTAGGG*, abrogates the activation of cytosolic nucleic acid-sensing cGAS and the AIM inflammasome by binding to these molecules in a manner that competes with immunostimulatory DNA and that A1515 decreases the volume of cerebral infarction and the number of dead cells by inhibiting the cGAS–STING signalling pathway in middle cerebral artery occlusion (MCAO) [17]. Interestingly, these researchers also found that knocking out the cGAS gene in microglia diminishes the protective effects of A151 in a cGAS-knockout rat model and a control rat model, which indicates close connections between cGAS and A151. In addition, some medicines that are already used in the clinic have been found to inhibit cGAS. For example, some antimalarial drugs, such as quinacrine, prevent the combination of cGAS and DNA and thus inhibit the cGAS–STING signalling pathway [118]. Unfortunately, the application of antimalarial drugs is not promising because the compounds are harmful to cells at a concentration of 10 µmol/L. It was found that aspirin acetylates cGAS at three lysine residues and blocks cGAS activity, which has an anti-inflammatory function in Trex1^−/−^ rats and fibroblasts from Aicardi–Goutieres syndrome (AGS) patients. However, the efficacy of these agents in CNS injuries is currently unknown and awaits further investigation [119]. Liu et al. found that epigallocatechin gallate (EGCG) indirectly blocks the interaction between cGAS and DNA by inhibiting the activity of GTPase-activating protein SH3 domain-binding protein 1 (G3BP1) [120]. However, its efficacy in CNS injury treatment remains unknown.

### Inhibitors of STING

Micronuclei [121], mtDNA [110], abnormal cell cycle [122], and cytoplasmic chromatin fragments [123] can activate STING in a cGAS-dependent manner. Several stimuli other than cGAMP, which is catalysed by cGAS, such as bacterial or virus cyclic dinucleotides (CDNs) [124, 125], can also directly activate STING. In addition, 1,25(OH) D regulates STING and IFNβ through a mechanism controlled by the hypoxia-inducible factor-1α (HIF-1α)–GATA1 axis [126]. The study provided the first demonstration that HIF-1α–GATA1 regulates STING. Thus, the targeted regulation of STING may exert a better therapeutic effect in CNS injury. STING is a type of transmembrane protein located in the ER. After binding 2′,3′-cGAMP, STING can induce the phosphorylation and dimerization of IRF-3, and activated IRF-3 then enters the cell nucleus and triggers expression of the IFN-I gene. Moreover, STING conveys signals to TNF receptor-associated factor 6 (TRAF-6), which induces the release of inflammatory factors such as TNF-a and IL-6 by activating the NF-κB signalling pathway [127]. Blockage of this link reduces the production of inflammatory factors and thus ameliorates the inflammatory reaction in response to CNS injury.

By screening compounds in vitro, researchers have identified a type of nitrofuran ramification called c-176. These researchers found that c-176 inhibits the ability of STING to combine with CDN by integrating with Cys-91 sites in rat STING, which blocks STING palmitoylation [61]. This procedure inhibits the cGAS–STING signalling pathway and markedly improves the progression of autoimmune disease. However, this compound has no effect on human STING [61]. Therefore, researchers have continued to study and screen H151 from a large number of compounds, and the results indicate that H151 covalently modifies human STING in the same manner as c-176.

Furthermore, some scholars have found that the invasion of viruses produces nitro-fatty acids (NO2-FAS), which combine with Cys88 and Cys91 residues in the N-terminal domain of STING through covalent bonds and thus prevent the palmitoylation of STING to inhibit its activation [128]. Some scholars have identified a compound that primarily acts on the mitochondrial fission mediator carbonyl cyanide 3-chlorophenylhydrozone (DRP-1-CCCP) [129]. These researchers found that CCCP does not affect the translocation of STING from the ER to the Golgi at the perinuclear region. However, they observed that the phosphorylation of STING was delayed (by 45 min) and diminished in CCCP-treated cells compared with vehicle-treated cells, which exhibited phosphorylated STING as early as 15 min posttreatment. In addition, CCCP can result in disruption of the mitochondrial membrane potential, leading to repressed communication between STING and TBK-1 [129]. Previous study has shown that mitochondrial fusion is pivotal to sustaining STING signalling pathway activation [130]. STING triggered by 2′,3′-cGAMP recruits and phosphorylates TBK-1 to its C-terminal tail (CTT) [130]. Phosphorylated TBK-1 then catalyses the phosphorylation of STING, which reduces the release of inflammatory factors such as IFN-I. In addition, Siu et al. screened a small-molecule inhibitor called compound 18, which combines with the C-terminal domain (CBD) pocket of STING and competes with 2′,3′-cGAMP to bind STING [131]. In human monocytes, compound 18 inhibits the expression of the IFN-B gene [131]. Li et al. discovered that AstinC, which was isolated from a natural plant called *Aster tataricus*, exerts STING-inhibiting activity by targeting the downstream transcription factor IRF-1 [132]. This compound was identified in a rat autoimmune model and shown to be effective in ameliorating rat autoimmune injury [132]. However, these medicines have not been used for CNS injury but are potential candidates for alleviating IFNs.

### Inhibitors upstream and downstream of cGAS–STING

Cytosolic DNA is linked to heightened inflammation, which is consistent with previous reports of increased levels of proinflammatory cytokines during ageing. Thus, the clearance of cytosolic DNA may afford therapeutic benefits. One of the pathways involved in the clearance of damaged or cytosolic DNA is autophagy, and defects in autophagy can potentiate the STING pathway and promote an inflammatory phenotype [85, 133]. Accordingly, the stimulation of autophagy can decrease the load of cytoplasmic DNA and afford therapeutic benefits [134]. For example, cytosolic chromatin accumulates within senescent cells. Cytosolic DNA fragments may also be derived from ruptured micronuclei or chromatin herniations, which are features of senescent cells [135, 136]. The researchers found that DNA fragments accumulate in senescent cells with activated cGAS–STING–NF-κB signalling and thus promote a senescence-associated secretory phenotype and cellular senescence. Intriguingly, we found that the metformin- or rapamycin-induced activation of autophagy significantly decreases the size and levels of DNA fragments and represses the activation of the cGAS–STING–NF-κB-senescence-associated secretory phenotype cascade and cellular senescence [134].

IFN-γ can stimulate JAK/STAT signalling pathways to enhance the inflammatory response. IFN-γ plays an important role in subsequent CNS injury. The suppression of downstream IFN-γ has also been explored as a therapeutic option. JAK1/2 inhibitors are neuroinflammation inhibitors, including ruxolitinib, which is widely used for various autoimmune/autoinflammatory conditions. Research has shown that ruxolitinib attenuates inflammatory responses by selectively inhibiting the JAK/STAT signalling pathway in IFN-γ-stimulated microglia, which leads to reductions in neuronal cell death and the inhibition of glial scar formation [137]. However, the immune responses in the CNS have dual effects, and wide immune suppression is more likely to yield side effects. Instead, optimal treatments should be tailored to augment the beneficial functions of neuroinflammation while simultaneously minimizing those that cause injury. As observed in clinical disease, baricitinib treatment improves the clinical manifestations and inflammatory and IFN biomarkers in patients with the monogenic interferonopathies CANDLE (chronic atypical neutrophilic dermatosis with lipodystrophy and elevated temperatures), SAVI (STING-associated vasculopathy with onset in infancy), and other interferonopathies [138].

## Conclusions and perspectives

CNS injury and its devastating consequences continue to challenge clinicians. Therefore, understanding the molecular basis of CNS injury may be beneficial for improving neuronal and glial survival and attenuating neurological deficits. Recent studies on STING signalling in the brain and spinal cord have increased our understanding of the role of this pathway in neural injury and neural innate immunity as well as in inflammation-mediated neurodegeneration.

Studies on the temporal pattern of STING activation in CNS injury are crucial and are beneficial for obtaining an understanding of STING and selecting appropriate time points for STING regulation after CNS injury. However, the results from these studies are not understood. As observed in a previous study using a mouse model, STING expression was increased at 2 h and at 24 h after TBI, and its expression at 24 h was higher than that at 2 h [49]. In the SAH model, the level of STING was significantly increased at 12 h after modelling, peaked at 24 h, and remained at a slightly high level until 72 h after injury [60]. However, these studies only investigated several limited time points in the short term. Further investigation is needed to elucidate the long-term temporal patterns and the changes in STING activity at multiple time points after injury. The chronic phase of CNS injury often occurs 3 days after injury, but the expression of STING after 3 days has not been studied. Neurodegeneration has a similar pathological process to the chronic phase of CNS injury, and previous studies have found that STING plays an important role in neurodegeneration (Parkinson’s and Alzheimer’s diseases). Targeting the cGAS–STING pathway can attenuate neuroinflammation and decrease senescence in cells and mainly has beneficial impacts on pathologies of neurodegenerative conditions [139, 140]. Based on the above-described studies, we speculate that STING may play a role in the chronic phase of CNS injury, which provides a direction for our future research on CNS injury.

STING activation occurs in response to a wide array of stressors, ranging from viral infection to ER and mitochondrial stress, which suggests that it is a major player in a number of neuropathologies. Because both beneficial and detrimental effects of STING having been reported, the targeting of this pathway is complex. In this review, we detail the current knowledge of the STING pathway and its protective and detrimental activity in acute CNS pathologies. In addition, we outline several potential links that warrant further investigation of the potential causal link between cGAS–STING and programmed cell death in CNS injury. We also explore potential drugs for the inhibition of STING activity and thus the treatment of CNS injury.

Based on these conclusions, we make some suggestions concerning future studies: (a) the correlation among the STING pathway and autophagy, necroptosis, ferroptosis, pyroptosis and lysosomal cell death in CNS injury should be comprehensively investigated; (b) it is essential to elucidate which type of programmed cell death plays the dominant role in CNS injury; (c) the regulatory mechanisms of the STING pathway in CNS injury, particularly methods to regulate the expression levels of programmed cell death-associated genes, should be explored; and (d) the relationship between miRNAs and the STING pathway is now well understood. Some research has found that miRNAs regulate the STING pathway [141, 142]. For example, miR-24–3p may ameliorate the inflammatory response and cellular apoptosis in the hepatic I/R process by targeting STING [141]. However, no study conducted to date has explored the effects of microRNAs on regulating the STING pathway in CNS injury. Therefore, studies investigating whether miRNAs affect the STING pathway in CNS injury are critical. (e) Other regulatory mechanisms, such as the anchoring and intracellular trafficking of STING, are also disrupted in CNS injury and remain unclear. Various other factors have been proposed to facilitate the export of STING or to anchor STING at the ER under stimulated conditions [37, 143]. However, whether STING anchoring and intracellular trafficking are disrupted in CNS injury has not been studied. Further research is needed. (f) The STING pathway plays an important role in senescence in neurodegenerative diseases, such as Parkinson’s and Alzheimer’s diseases. One study reported that cGAS–STING activation was elevated in mice with Alzheimer’s diseases. Targeting the cGAS–STING pathway can attenuate neuroinflammation, decrease senescence in cells and ultimately improve learning, memory and synaptic plasticity in mice with Alzheimer’s disease [144]. However, the relationship between STING and ageing in CNS injury has not been explored and will be a very meaningful research topic.

Although the STING pathway plays important roles in different types of programmed cell death, we stress that the complexity of CNS injury cannot be reduced to a single pathophysiological mechanism or to the inhibition or activation of a single molecular pathway. Therefore, specific combination therapies targeting the cGAS–STING pathway may represent a promising strategy for the treatment of CNS injury. Furthermore, the translation of the current findings to the postinjury administration of drugs that block or activate the cGAS–STING pathway needs further effort. In conclusion, this review can help elucidate the functions of the STING pathway in the pathological process of CNS injury. Thoughtful consideration and more detailed findings concerning the roles of the STING pathway will contribute to improving our understanding of CNS injuries of unknown aetiology in the near future.

## Data Availability

Not applicable.

## Abbreviations

STING: Stimulator of interferons genes
CNS: Central nervous system
SCI: Spinal cord injury
TBI: Traumatic brain injury
SAH: Subarachnoid haemorrhage
HI: Hypoxia–ischaemia
cGAS: Cytosolic DNA sensor cyclic GMP-AMP synthase
IFN: Interferon
NF-κB: Nuclear factor κB
dsDNA: Double-stranded DNA
ER: Endoplasmic reticulum
IRF3: Interferon regulatory factor 3
CDNs: Cyclic dinucleotides
PAMPs: Pathogen-related molecule patterns
DAMPs: Danger-related molecule patterns
PRRs: Pattern-recognition receptors
TLR9: Toll-like receptor 9
AIM2: Absent in melanoma 2
IL-1β: Interleukin-1β
IL-18: Interleukin-18
2′,3′-cGAMP: 2',3'-Cyclic AMP-GMP
ATP: Adenosine triphosphate
GTP: Guanosine triphosphate
STIM1: Sensor stromal interaction molecule
COPII: Coat protein complex II
ERGIC: ER–Golgi media compartment
TBK1: TANK-binding kinase 1
CCI: Controlled cortical impact
RIPK1: Receptor-interacting protein kinase 1
LINE-1: Long-interspersed element-1
MAPKs: Mitogen-activated protein kinases
ISGs: Interferon-stimulated genes
PCD: Programmed cell death
ROS: Reactive oxygen species
GPX4: Glutathione peroxidase 4
ASC: Adaptor protein apoptosis-associated speck-like protein
GSDMD: Gasdermin D
